# Bacterial Adhesion and Biofilm Formation of *Enterococcus faecalis* on Zwitterionic Methylmethacrylat and Polysulfones

**DOI:** 10.3389/fcimb.2022.868338

**Published:** 2022-05-16

**Authors:** Franziska Woitschach, Marlen Kloss, Karsten Schlodder, Alexander Borck, Niels Grabow, Emil Christian Reisinger, Martina Sombetzki

**Affiliations:** ^1^ Division of Tropical Medicine and Infectious Diseases, Center of Internal Medicine II, University Medical Center, Rostock, Germany; ^2^ Biotronik SE & Co. KG, Research & Development, Berlin, Germany; ^3^ Institute for Biomedical Engineering, University Medical Center Rostock, Rostock, Germany

**Keywords:** biofilm, *Enterococcus faecalis*, zwitterion, methylmethacrylat, polysulfone

## Abstract

Biofilm-associated implant infections represent a major challenge for healthcare systems around the world due to high patient burden and enormous costs incurred. *Enterococcus faecalis (E. faecalis)* is the most prevalent enterococcal species identified in biofilm-associated infections. The steadily growing areas of application of implants demand a solution for the control of bacterial infections. Therefore, the development of modified anti-microbial implant materials and the testing of the behavior of different relevant bacterial strains towards them display an indispensable task. Recently, we demonstrated an anti-microbial effect of zwitterionic modified silicone rubber (LSR) against *Staphylococcus aureus*. The aim of this study was to evaluate bacterial colonization and biofilm formation of another clinically relevant strain, *E. faecalis*, on this material in comparison to two of the most commonly used thermoplastic polyurethanes (TPUs) and other modified LSR surfaces. By generating growth curves, crystal violet, and fluorescence staining, as well as analyzing the expression of biofilm-associated genes, we demonstrated no anti-microbial activity of the investigated materials against *E. faecalis*. These results point to the fact that anti-microbial effects of novel implant materials do not always apply across the board to all bacterial strains.

## Introduction

The ability of bacteria to form biofilms on foreign bodies is an important virulence factor, as bacteria in a biofilm can remain in the human body for a long time, and are resistant to both, immune system defense and antibiotic treatment. The most common pathogens in these cases are staphylococci and enterococci. One representative of enterococci is *E. faecalis*, a common gram-positive germ of nosocomial and community-acquired infections associated with high morbidity and mortality (Law et al., 1994), with endocarditis being one of the most serious (Fernández Guerrero et al., 2007).

In general, bloodstream infections in 7% of European and 10% of US care units are caused by enterococci (Fridkin and Gaynes, 1999; Fluit et al., 2001). The biofilm formation capability of *E. faecalis* has recently been suggested as an important factor in the pathogenesis of enterococci infections (Sandoe et al., 2002; Sandoe et al., 2003; Ramadhan and Hegedus, 2005). Like many other pathogenic microorganisms, *E. faecalis* adheres efficiently to biotic and abiotic surfaces, secreting a protective extracellular matrix that leads to the formation of a multilayer antibiotic-resistant biofilm (Dunny et al., 2014). This intrinsic antibiotic resistance poses a serious challenge for the treatment of enterococci infections (Suriyanarayanan et al., 2018). Bacterial adhesion and the resulting biofilm formation on implants are strongly dependent on their surface properties. Therefore, optimization of implant surfaces with regard to anti-adhesion is crucial. Two main strategies exist to inhibit microbial colonization on surfaces: the use of materials with anti-microbial or anti-adhesive properties (Shi et al., 2008; Zhang et al., 2008; Lönn-Stensrud et al., 2009). An anti-microbial surface structure can be achieved by functionalization of the material with bactericidal substances, such as the incorporation of antibiotics. Anti-adhesive conditions can be achieved by using polymers (Kingshott et al., 2003), such as polymethylmethacrylat (PMMA), preventing attachment of bloodstream bacteria. PMMA is used in various medical fields due to its excellent biocompatibility (Frazer et al., 2005). However, the properties of anti-adhesive materials are often not sufficient to prevent bacterial adhesion. Subsequently, the development of novel functionalization strategies is needed. The optimization with zwitterions is a promising approach to reduce the initial attachment of proteins to the material (Azari and Zou, 2013). One of the best characterized zwitterionic material is 2-methacryloyloxylethyl phosphorylcholine (MPC). Another example for functionalization was implemented by protein repellant polysulfone (PSU) and poly(1,4-phenylene-ether-ether-sulfone) (PPSP), which are often used to produce membranes for ultrafiltration (Yunos et al., 2014) due to their hydrophobicity, high pH- and temperature resistance.

In our previous work, we investigated the biofilm formation ability of two different staphylococcal strains on liquid silicone rubber (LSR) surfaces modified with PMMA-MPC, PSU, and PPSP compared to two of the most commonly used thermoplastic polyurethanes (TPU). We found an inhibitory effect for *Staphylococcus aureus* (ATCC35556) on LSR coated with PMMA-MPC (Woitschach et al., 2021a). In this study, we would like to extend our protocol for analyzing bacterial colonization and biofilm formation on these materials by *Enterococcus faecalis*.

## Materials and Methods

### Zwitterionic Materials and controls

We assessed the infection resistance of different implant materials after confrontation with *E. faecalis* by comparing them using various analyses. The materials were kindly provided by Biotronik (Berlin). Two thermoplastic polyurethanes (TPU) and liquid silicone rubber (LSR) were used, either with or without modification. Further details on the preparation are described in our previous work (Woitschach et al., 2021a; Woitschach et al., 2021b). After modification, all samples were subjected to a sterilization process with ethylene oxide (ETO). Thermanox, a well-established polyester, was used as a reference. All materials were round and had a diameter of approximately 16 mm. The following abbreviations were used: Thermanox = tmx, Thermoplastic polyurethane 55 = P55, Thermoplastic polyurethane 80 = P80, unmodified Liquid silicone rubber = LSR, LSR + Polymethylmethacrylate-2-methacryloyloxyethyl phosphorylcholine = PMMA-MPC, LSR + Polysulfone = PSU and LSR + Poly (1,4-phenylene-ehter-ether-sulfone) = PPSP.

### Bacterial Strain and Culture Conditions

Snap frozen *Enterococcus faecalis* (ATCC29212) was purchased from the American Type Culture Collection (ATCC). *E. faecalis* was grown in brain heart infusion broth (BHI) supplemented with 1% glucose (Kwasny and Opperman, 2010). For all experiments an inoculum with OD_600_ of 0.1 (optical density measured with wavelength 600 nm) was prepared by adjusting the concentration of an overnight bacterial broth culture in BHI medium, this corresponds to 1 x 10^8^ CFU/ml (colony forming unit/ml). All experiments were carried out aerobically at 37°C in 24-well polystyrene plates (Nunc, Thermofisher scientific, Germany) with a culture volume of 2 ml unless otherwise stated and were performed in three independent replicates.

### Bacterial Viability

Bacterial viability was analyzed by generating growth curves using OD measurement and performing LIVE/DEAD fluorescent staining. In brief, 2 ml of the bacterial culture (1 x 10^7^ CFU/ml) were added to a 24-well plate containing the different rounded material disks and incubated for 6 h. Bacterial growth was monitored hourly by measuring OD_600_ using a microplate reader (FLUOstar Omega, BMG Labtech). In addition, bacterial viability was determined after 6 and 24 h using the two-color fluorescence assay LIVE/DEAD™ BacLight™ Bacterial Viability Kit (Thermo Fischer, Waltham, Massachusetts, USA) according to the manufacturer’s instructions. The fluorescence was visualized using a fluorescence microscope (Axio Scope.A1, Zeiss, Germany) equipped with a camera (AxioCam MRc, Zeiss, Jena, Germany). The fluorescence intensity was determined using a microplate reader (FLUOstar Omega, BMG Labtech) to subsequently calculate the percentage of living cells using the adjusted dye ratio equation as follows: % living cells = (100 x SYTO^®^/PI)/(1+ SYTO^®^/PI) in accordance to Ou et al. (2016.). SYTO^®^ 9 green-fluorescent nucleic acid (SYTO^®^) stain labels all bacteria in a population (live) and the red-fluorescent nucleic acid stain, propidium iodide (PI), is taken up by bacteria with damaged membranes (dead) only.

### Material Toxicity

The disc diffusion test was used to determine the inhibition of bacterial growth by substances released by the materials. The agar plates were inoculated with 1 x 10^8^ CFU/ml of the bacterial culture. The different material discs were then placed in the middle of the inoculated agar surfaces. The plates were incubated overnight at 37°C and the diameters of the inhibition zones around the discs were measured.

### Bacterial Cell Surface Hydrophobicity

Using the MATH/BATH test (Microbial Adhesion to Hydrocarbons/Bacterial Adherence to Hydrocarbons), the hydrophobicity of the bacterial cell surface was assessed according to Lather et al. (2016) and Di Ciccio et al. (2015), with minor modifications. For this purpose, the bacteria were centrifuged at 3000 g for 5 min. The pellet was washed three times in ice-cold phosphate buffer and resuspended in a specific amount of phosphate buffer to achieve an OD_500_ of 0.5. The bacterial suspension was overlaid with 0.5 mL of xylene (Sigma Aldrich, Darmstadt, Germany). After shaking for 1 min, the phases were incubated at room temperature for 15 min for separation. Results were expressed as the percentage of cells excluded from the aqueous phase as determined by the following equation: % adherence = [(1 -*A*/*A*0)] × 100, where A0 and A are the initial and final optical densities of the aqueous phase, respectively. The classification was made as follows: >50% = strongly hydrophobic; 20 - 50% = moderately hydrophobic; <20% = hydrophilic. The measurements were performed twice with three technical replicates.

### Biofilm Quantification

Crystal violet staining was used to quantify biofilm formation on the materials, as previously described (Kwasny and Opperman, 2010). Briefly, 2 ml of the bacterial suspension (1 x 10^8^ CFU/ml) was incubated on the materials for 24 h. The supernatant was removed, and the discs were washed three times with double-distilled water to remove bacteria that were not or were only loosely attached. The discs were incubated for 1 h at 60°C for heat fixation. 200 µl of 0.06% crystal violet was added to each well and incubated for 5 min. The crystal violet was then removed and the slices were washed three times with double distilled water. Stained biofilms on the discs were eluted with 200 µl of 30% acetic acid. The supernatants were measured at 600 nm in a multilabel microtitre plate (FLUOstar Omega, BMG Labtech).

### Gene Expression Quantification

Isolation and purification of mRNA was carried out using the Fungal/Bacterial RNA Miniprep Kit (Zymo Research, California, USA). The mRNA was extracted from *E. faecalis* biofilms after 6 and 24 h and quantified by spectrophotometric measurement (Colibri, Berthold Technologies, Bad Wildbad, Germany). An amount of 500 ng of total RNA was transcribed using High-Capacity cDNA Reverse Transcription Kit (ThermoFisher, Dreieich, Germany), according to the manufacturer’s instructions. Gene expression levels were determined using two-step quantitative reverse transcription PCR with 50 ng of cDNA in a reaction volume of 10 µl using TaqMan Universal MasterMix II (ThermoFisher, Dreieich, Germany). The primers used in this study were selected with regard to their involvement in biofilm formation: Gelatinase (*GelE*) F: 5’-GGTGACCCCGTATCATTGGT-3’ R: 5’-CCGTGTAAAGCAATTCCCGT-3’ Internal Oligo: 5’-ACGGAACATGTCGGTAGTGA-3’; vWF-binding protein (*EbpA*) F: 5’-CGCCAAGTAGCACTAGAGGA-3’ R: 5’-GTCCCGTAAAAGCGACCATC-3’ Internal Oligo: 5’-GTCGAGTTCAAACAGAGGCG-3’; LPXTG-anchored aggregation substance (*AS*) F: 5’-AGTCAAAATGGCGCCACAAT-3’ R: 5’-CAAACACAGACCATTGGCCA-3’ Internal Oligo: 5’-TACGCTGGTGTTGTGGAAGA-3’; *Enterococcus faecalis* antigen A (*EfaA*) F: 5’-TAGAAACAGGCGGAAATGGC-3’ R: 5’-AACCAAGCATGCGGATCTTC-3’ Internal Oligo: 5’-CAAGTGCCGGTCAAGAACAA-3’. 16S was used as an endogenous control F: 5’-GGGAATCTTCGGCAATGGAC-3’ R: 5’-CGCCCAATAAATCCGGACAA-3’ Internal Oligo: 5’-GAAAGTCTGACCGAGCAACG-3’. Primers and probes were purchased from Eurofins Genomics, Germany. Relative fold change in gene expression was determined using the 2^−ΔΔct^ method. Data were presented as the fold-change to tmx normalized to the reference gene expression level of 16S.

### Statistics

Statistical analysis was performed using GraphPad Prism 5.0 (GraphPad Software, La Jolla, CA, USA). Values are expressed as mean + SEM. Normal distribution was tested using the D’Agostino and Pearson Omnibus Normality Test. Non-normally distributed samples were compared using the Kruskal–Wallis test followed by a Dunn’s *post hoc* test. For all statistical analyses, *p* values < 0.05 were considered significant. *p < 0.05, **p < 0.01, ***p < 0.001, n.s. = not significant. Tmx served as control material.

## Results

### Growth Effects and Biofilm Formation of *Enterococcus faecalis* on Thermoplastic Polyurethanes and Modified Silicone Rubbers

**Figure 1:**
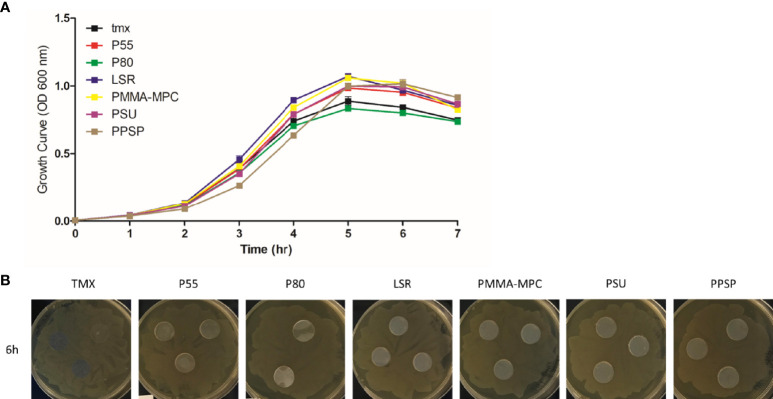
The effects of the investigated surface modifications of the materials on *E. faecalis* growth. **(A)** Growth behavior of *E. faecalis* (ATCC 29212) was assessed at 37°C with shaking at 150 rpm in liquid BHI medium on the different material disks. Growth was monitored hourly by measuring OD_600_. Results are presented as averages + SEM of three independent experiments performed in duplicates. **(B)** Investigations of inhibition of bacterial growth by substances released by the material were performed using disk diffusion test. 1 x 10^8^ CFU/ml of the bacterial culture were seeded on agar plates. The different material disks were placed in the middle of the plates and incubated at 37°C for 6 h Representative images of the plates with the different materials are shown.

The growth curves, which represent the density of cell populations in liquid culture over time, showed a comparable bacterial growth on all materials tested, represented by an increase in the first 5 h up to an OD of approximately 1.0, followed by a slight decrease (**Figure 1A**). To investigate whether inhibitory substances are released by the materials, we performed a disc diffusion test. The absence of inhibition zones indicated the non-toxicity of the materials (**Figure 1B**).

**Figure 2:**
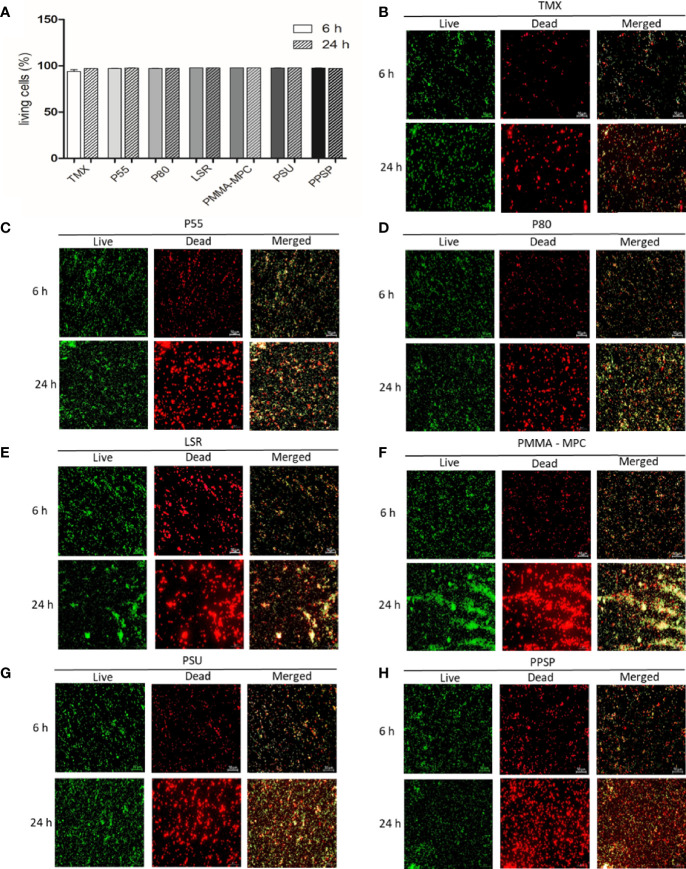
Viability and biofilm development of *E. faecalis* at two different time points. **(A)** After 6 and 24 h, bacterial viability was determined using LIVE/DEAD™ BacLight™ Bacterial Viability Kit two-color fluorescence assay. Fluorescence intensity was measured using a microplate reader (FLUOstar Omega, BMG Labtech) to calculate the percentage of living cells using the adjusted dye ratio equation as follows: % living cells = (100 x SYTO^®^/PI)/(1+ SYTO^®^/PI) in accordance to Ou et al. (2016). Results are presented as averages of three independent experiments with two technical replicates + SEM. **(B–H)** The fluorescence was visualized using a fluorescence microscope (Axio Scope.A1, Zeiss, Germany) equipped with a camera (AxioCam MRc, Zeiss, Jena, Germany). SYTO^®^ 9 green-fluorescent nucleic acid (SYTO^®^) stain labels all bacteria in a population (live) and the red-fluorescent nucleic acid stain, propidium iodide (PI), penetrates only bacteria with damaged membranes (dead). The scale for all images shown is 50 µm.

The ability of the materials to affect the viability of *E. faecalis* was analyzed using the LIVE/DEAD™ BacLight™ Bacterial Viability Kit. *E. faecalis* was seeded for 6 and 24 h on the different materials. The percentage of living bacteria was calculated as mentioned above. We obtained more than 95% living cells for at both time points with no discernible differences between the various materials (**Figures 2A**). In addition, the fluorescence microscope images of *E. faecalis* showed that the surface of the discs was covered by nearly the same amounts of adhered living bacteria (green) and dead bacteria (red) on all materials (**Figures 2B–H**).

**Figure 3:**
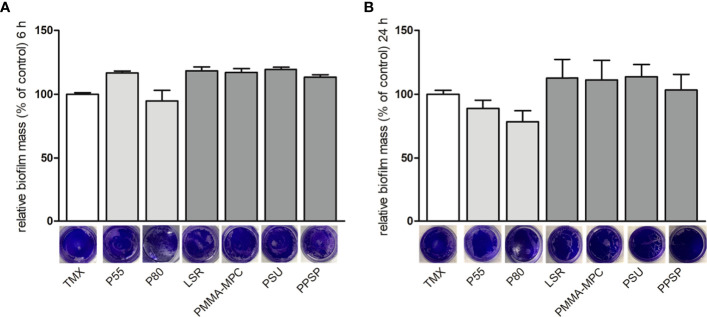
The effects of the investigated surface modifications of the materials on *E. faecalis* biofilm formation. *E. faecalis* (1 x 10^8^ CFU/ml) was cultured on the different material discs and incubated at 37°C for 6 and 24 h. Biofilm formation was assessed by crystal violet staining. Results are presented as averages of three independent experiments + SEM. **(A)** 6 and **(B)** 24 h.

Bacterial biofilm formation was quantified by crystal violet staining. After 6 and 24 h no pronounced differences were observed in terms of biofilm formation on the tested materials, as evidenced by the relatively similar intensity of staining, except for a slight decrease on P80 at both time points, as well as for P55 after 24 h (**Figures 3A, B**).

**Figure 4:**
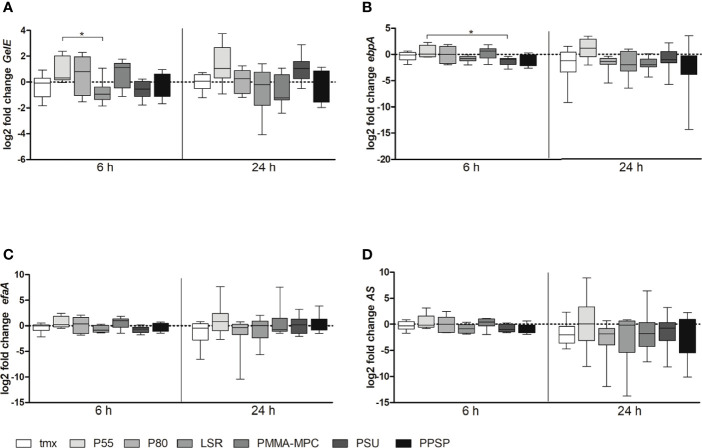
The mRNA expression levels of biofilm-associated genes of *E. faecalis. E. faecalis* (1 x 10^8^ CFU/ml) was cultured on the different material discs and incubated at 37°C for 6 and 24 h. Relative gene expression of **(A)** *GelE*, **(B)** *ebpA*, **(C)** *efaA* and **(D)** *AS* was determined by real-time PCR. Data are represented as mean + SEM of 6 independent experiments. Significant results are indicated as *p < 0.05.

Also, we analyzed biofilm formation relevant gene expression of *GelE, ebpA, efaA* and *AS* after 6 and 24 h. We found a tendency towards lower expression levels for *GelE* after 6 h for all materials except of PMMA-MPC. This effect disappeared at later time points and, in terms of PMMA-MPC, even opposite gene expression level was reached (**Figure 4A**). In addition, for *GelE* significant differences between P55 and LSR, with an approximate reduction by half after 6 h (**Figure 4A**) and for *ebpA* between P55 and PSU (**Figure 4B**) were determined.

For further characterization of *E. faecalis*, we analyzed the hydrophobicity of the bacterial cell surface by the MATH/BATH (Microbial Adhesion to Hydrocarbons/Bacterial Adherence to Hydrocarbons) assay. Analysis of the hydrophobic properties of the cell surface of *E. faecalis* revealed with 39.74 ± 14.73% only moderate hydrophobicity.

## Discussion

In the present study, we report the analysis of two different commonly used TPUs (P55, P80), unmodified LSR and three modified LSRs (PMMA-MPC, PSU, PPSP) for their anti-bacterial potential against *E. faecalis* colonization. The results of our study reveal that there is no inhibitory effect of the materials on the viability or biofilm formation for *E. faecalis*, which is also confirmed by gene expression. In our previous work, we were able to demonstrate that the modified LSR with PMMA-MPC exhibited resistance to *Staphylococcus aureus* adhesion and biofilm formation (Woitschach et al., 2021a). We provided strong evidence that the inhibition of *S. aureus* biofilm formation was due to the surface attraction properties of PMMA-MPC, which is mainly attributed to composition of surface free energy (Woitschach et al., 2021a). In the present study, neither qPCR, LIVE/DEAD staining, nor crystal violet showed evidence of reduced biofilm formation of *E. faecalis* on this material. This is consistent with a study by Velreads et al. pointing to the ability of *E. faecalis* to adhere to hydrophobic silicone rubber surfaces (Velraeds et al., 1997). No inhibitory effect could be detected for the more hydrophilic material PMMA-MPC either. The fact that *E. faecalis* is only moderately hydrophobic, as shown by the MATH/BATH test, could be an indication of this, as hydrophilic cells attach more easily to hydrophilic surfaces (Kochkodan et al., 2008; Giaouris et al., 2009). In this regard, we have shown in our previous study that PMMA-MPC is more hydrophilic than the other materials used (Woitschach et al., 2021a). Another study also reported that *E. faecalis* has a strong intercellular attraction. This is due to certain proteins, for example sex pheromones, which are increasingly expressed after interaction with other bacterial cells (Waar et al., 2002). Furthermore, it is known that the interaction force between cells adhering to each other is higher compared to cells and substrate (van der Mei et al., 1993). These may also be processes that account for the ineffectiveness of the materials against *E. faecalis* colonization. In summary, considering the methodology used and the results obtained, we investigated that *E. faecalis* was able to develop biofilm on all the materials tested during incubation periods of 6 and 24 h. Considering the age of antibiotic resistance, it is highly relevant to explore innovative strategies for bacterial defense. Especially with regard to bacterial properties, for example surface characteristics and virulence factors, the material features have to be adapted. And in terms of bacterial diversity, it is equally important to test promising strategies against the area-specific pathogens to uncover any loopholes.

## Funding

This study was financially supported by the Federal Ministry of Education and Research (BMBF) with RESPONSE “Partnership for Innovation in Implant Technology”.

## Conflict of Interest

Authors KS and AB are employed by Biotronik.

The remaining authors declare that the research was conducted in the absence of any commercial or financial relationships that could be construed as a potential conflict of interest.

## Publisher’s Note

All claims expressed in this article are solely those of the authors and do not necessarily represent those of their affiliated organizations, or those of the publisher, the editors and the reviewers. Any product that may be evaluated in this article, or claim that may be made by its manufacturer, is not guaranteed or endorsed by the publisher.
